# Crystal structures of two mixed-valence copper cyanide complexes with *N*-methyl­ethylenedi­amine

**DOI:** 10.1107/S2056989017000111

**Published:** 2017-01-10

**Authors:** Peter W. R. Corfield, Alexander Sabatino

**Affiliations:** a Department of Chemistry, Fordham University, 441 East Fordham Road, Bronx, NY 10458, USA

**Keywords:** crystal structure, mixed valence copper complex, *N*-methyl­ethylenedi­amine, cyanide, three-dimensional polymer, cuprophilic inter­actions

## Abstract

The crystal structures of two mixed-valence copper cyanide compounds involving *N*-methyl­ethylenedi­amine (meen), are described. In compound (I), poly[bis(μ_3_-cyanido-κ_3_ *C*:*C*:*N*)tris(μ_2_-cyanido-κ_2_ *C*:*N*)bis(*N*-methylethane-1,2-di­amine-κ_2_ *N*,*N*′)tricopper(I)copper(II)], [Cu_4_(CN)_5_(C_3_H_10_N_2_)_2_] or Cu_4_(CN)_5_meen_2_, cyanide groups link Cu^I^ atoms into a three-dimensional network containing open channels parallel to the *b* axis. In the network, two tetra­hedrally bound Cu^I^ atoms are bonded by the C atoms of two end-on bridging CN groups to form Cu_2_(CN)_6_ moieties with the Cu atoms in close contact at 2.560 (1) Å. Other trigonally bound Cu^I^ atoms link these units together to form the network. The Cu^II^ atoms, coordinated by two meen units, are covalently linked to the network *via* a cyanide bridge, and project into the open network channels. In the mol­ecular compound (II), [(*N*-methylethylenediamine-κ^2^ *N*,*N*′)copper(II)]-μ^2^-cyanido-κ^2^ *C*:*N*-[bis(cyanido-κ*C*)copper(I)] monohydrate, [Cu_2_(CN)_3_(C_3_H_10_N_2_)_2_]·H_2_O or Cu_2_(CN)_3_meen_2_·H_2_O, a CN group connects a Cu^II^ atom coordinated by two meen groups with a trigonal–planar Cu^I^ atom coordinated by CN groups. The mol­ecules are linked into centrosymmetric dimers *via* hydrogen bonds to two water mol­ecules. In both compounds, the bridging cyanide between the Cu^II^ and Cu^I^ atoms has the N atom bonded to Cu^II^ and the C atom bonded to Cu^I^, and the Cu^II^ atoms are in a square-pyramidal coordination.

## Abstract 2

Two mixed-valence compounds have been isolated from copper-cyanide-meen systems, Cu_4_(CN)_5_meen_2_ and Cu_2_(CN)_3_meen_2_·H_2_O, where meen is *N*-methyl­ethylenedi­amine. The former crystallizes as a polymer, in which Cu^II^meen_2_ moieties are covalently linked *via* cyanide bridges to a three-dimensional Cu^I^ cyanide-bridged array, while the latter is a binuclear monomer.

## Chemical context   

There is continuing inter­est in the synthesis and structures of coordination polymers involving CuCN networks (Etaiw *et al.*, 2015 ▸, 2016 ▸; Cai *et al.*, 2011 ▸). The structure determinations described here arise from our ongoing syntheses of mixed-valence copper cyanide complexes incorporating various amines, with the aim of the directed synthesis of new polymeric structures. A variety of crystal structures form from Cu^I,II^-cyanide-multidentate amine systems, ranging from the classic three-dimensional mixed-valence structure Cu_3_(CN)_4_en_2_·H_2_O where en is ethyl­enedi­amine (Williams *et al.*, 1972 ▸) to mol­ecular compounds such as Cu_2_(CN)_3_eten_2_ (Corfield & Michalski, 2014 ▸), where eten is *N*-ethyl­ethylenedi­amine. Syntheses involving *N*-methyl­ethylenedi­amine, meen, led to the formation of blue crystals of (II), Cu_2_(CN)_3_meen_2_·H_2_O, which formed as elongated plates. Their structure described here is that of a mol­ecular compound very similar to the eten derivative referred to above. Syntheses with meen have also been carried out in the presence of tetra­hedral monovalent anions such as BF_4_^−^ and ClO_4_^−^, in the hope that incorporation of negative ions might induce crystallization of a polymeric structure. The major or sometimes sole product in these preparations were well-formed polyhedral black crystals of (I), Cu_4_(CN)_5_meen_2_, which we found to indeed be made up of a three-dimensional network, but, somewhat to our surprise, without any incorporation of BF_4_^−^ or ClO_4_^−^ anions.
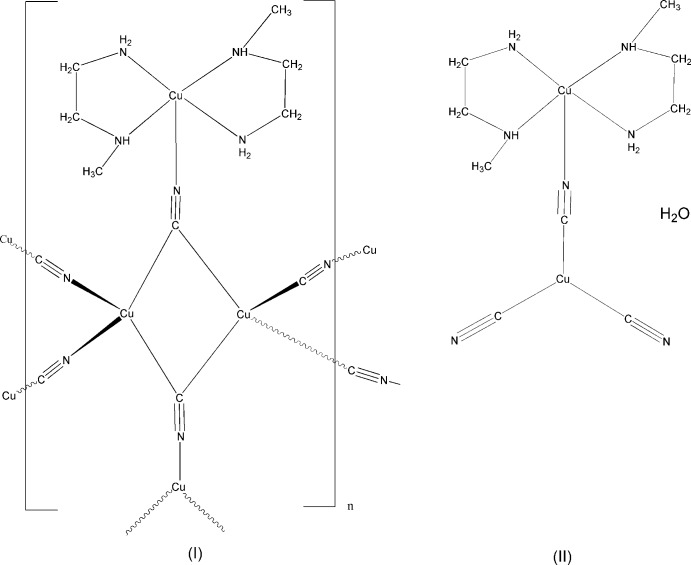


## Structural commentary   

**Figure 1:**
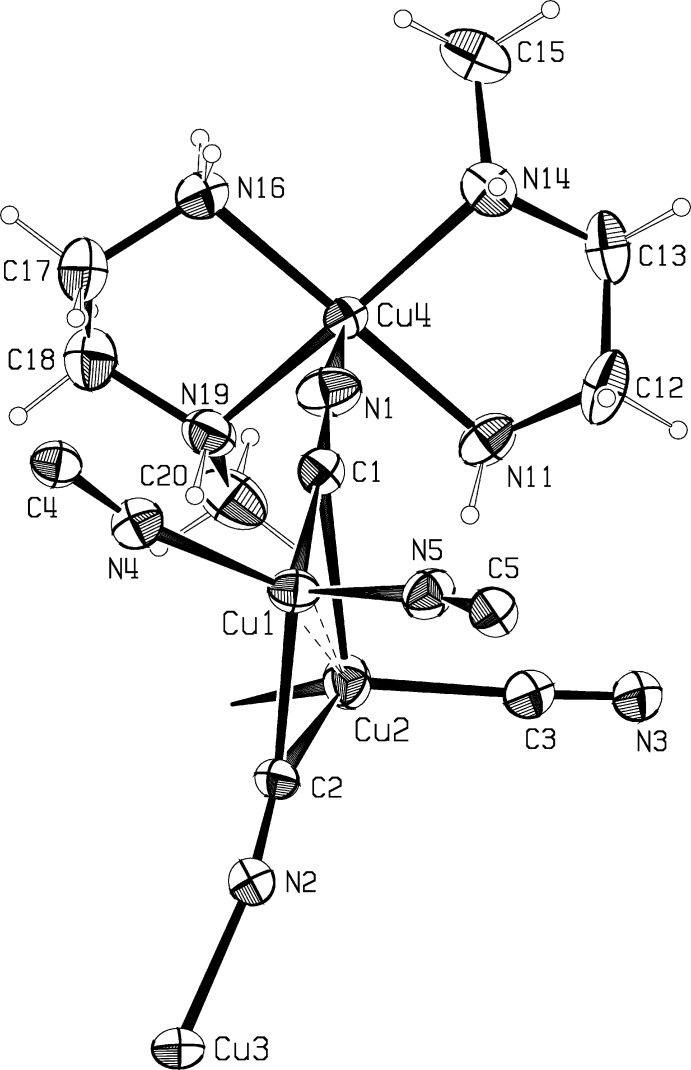
The asymmetric unit for compound (I), Cu_4_(CN)_5_meen_2_. Ellipsoids are drawn at the probability 50% level. The cuprophilic inter­action is shown as a dashed bond.

**Figure 2:**
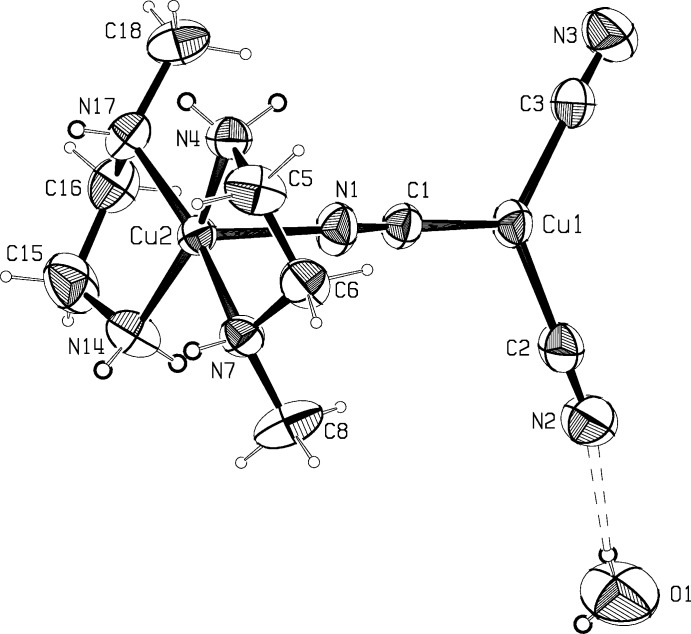
The asymmetric unit for compound (II), Cu_2_(CN)_3_meen_2_·H_2_O. Ellipsoids are drawn at the probability 50% level. The refined N- and O-bound hydrogen atoms are emphasized.

The asymmetric units for compounds (I) and (II) are shown in Fig. 1 ▸ and Fig. 2 ▸. Compound (I), Cu_4_(CN)_5_meen_2_, crystallizes as a three-dimensional cyanide-bridged Cu^I^ network, with Cu^II^meen_2_ units covalently anchored to the network *via* the bridging C1—N1 group, with N1 bonded to Cu^II^. The network is assembled from Cu_2_(CN)_6_ units, trigonal–planar Cu(CN)_3_ units, and square-pyramidal Cumeen_2_(CN) units. Compound (II) crystals contain monomeric dinuclear mol­ecules of Cu_2_(CN)_3_meen_2_·H_2_O.

The dimeric Cu_2_(CN)_6_ units in (I) are comprised of tetra­hedrally coordinated atoms Cu1 and Cu2 held closely together by two μ_3_-CN groups coordinating to both Cu atoms *via* C1 and C2, with short Cu ⋯ Cu distances of 2.560 (1) Å. The Cu—C and Cu—N distances listed in Table 1 ▸ show that the end-on CN bridging is not symmetrical, with Cu1—C1 and Cu1—C2 distances of 0.2–0.3 Å less than the corresponding bond lengths to Cu2. This asymmetry is the norm for such dimers, as noted in Corfield *et al.* (2016 ▸). The assumed cuprophilic attraction distorts the tetra­hedral coordination around Cu1, with the C1—Cu1—C2 angle opened up to 118.01 (13)°, while the N4—Cu1—N5 angle opposite is reduced to 102.67 (12)°. The situation is reversed for Cu2, where the C1—Cu2—C2 angle is 96.13 (11)° and the opposite angle C3—Cu2—C4 is increased to 128.27 (13)°.

Trigonal coordination at Cu3 in (I) is distorted, with angles ranging from 112.56 (12)° to 129.79 (13)°; the coordination is rigidly planar, however. In (II), coordination at the trigonal planar Cu^I^ atom is much more regular, with angles ranging from 117.49 (7)° to 122.15 (7)°.

Both (I) and (II) contain Cu^II^ atoms coordinated by two bidentate meen ligands and the N atom of a bridging cyanide group, in square-pyramidal coordination. Cu atoms are 0.122 (1) and 0.220 (1) Å from the best plane through the amine N atoms in (I) and (II), respectively. In (I), the Cu—NH(CH_3_) bonds are 0.05–0.07 Å longer than the Cu—NH_2_ bonds (Table 1 ▸), whereas the corresponding bond lengths are more similar in (II)(Table 2 ▸), as also seen in the *N*-ethyl complex corresponding to (II), Cu_2_(CN)_3_eten_2_ (Corfield & Michalski, 2014 ▸).

Coordination of the methyl­ated N atom in meen to Cu4 produces a chiral center. N atoms in the (*x,y,z*) atoms of (I) have the *R* configuration, and the chelate rings have the δ conformation, with N—C—C—N torsion angles of 54.6 (4)° and 56.0 (4)°. Glide-plane-related rings will have the *S*λ combination. Methyl­ated N atoms in the Cumeen_2_ units in (II) both have the *SS*δδ configuration, with N—C—C—N torsion angles of −53.0 (2)° and −53.1 (2)°. The center of inversion in (II) causes an equal number of mol­ecules with the *RR*λλ combinations. The CH_3_—N—C—C torsion angles in the chelate rings depend on the *R*/*S* and δ/λ combination. For an *R*δ combination, this angle will be approximately −170°, and for *R*λ the angle will be about −90°. These angles are reversed in sign for the *S*λ and *S*δ combinations. CH_3_—N—C—C angles are −172.8 (3) and −167.4 (3)° in (I), and 175.0 (2) and 174.5 (2)° in (II). Averages for these angles in 24 Cumeen chelate rings are reviewed in the Database Survey section.

## Supra­molecular features   

**Figure 3:**
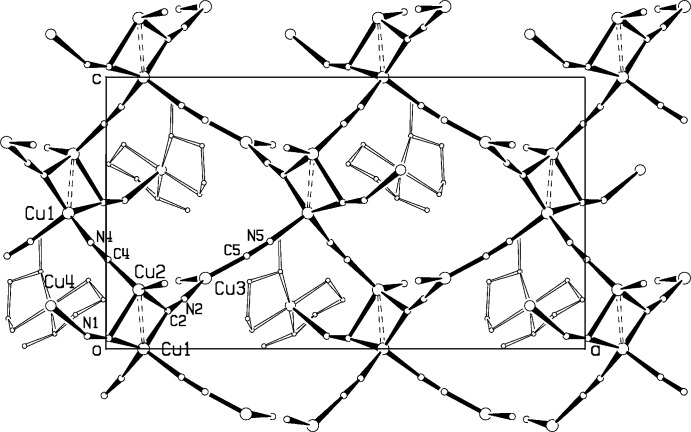
Packing diagram for compound (I), Cu_4_(CN)_5_meen_2_, giving a projection down the *b* axis. Cuprophilic inter­actions are shown as dashed bonds. For clarity, only a section of the structure perpendicular to *b* is shown.

**Figure 4:**
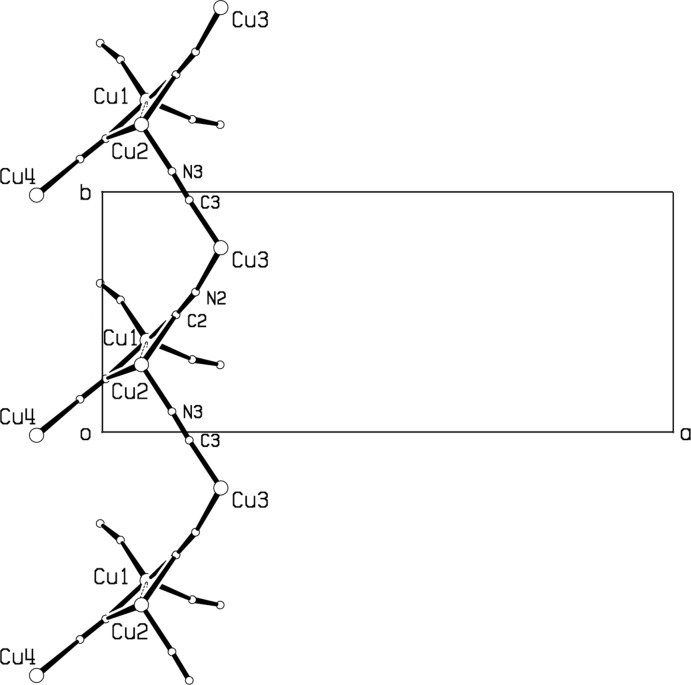
Projection of part of the structure of compound (I), Cu_4_(CN)_5_meen_2_ down the *c* axis, showing a chain along the *b* direction. Cuprophilic inter­actions are shown as dashed bonds.

In (I), each dimeric Cu_2_(CN)_6_ unit is linked by the C4–N4 cyanide group to a screw-related Cu_2_(CN)_6_ unit to form chains of these units parallel to the *c* axis, Fig. 3 ▸. Trigonally coordinated Cu3 also links the Cu_2_(CN)_6_ units together via CN bridges into single-stranded chains along the 8.231 (1) Å *b* axis, Fig. 4 ▸, similar to the double-stranded chains along the 8.356 (1) Å *a* axis seen in the polymeric compound (et_2_oenH)[Cu_2_(CN)_3_], (Corfield *et al.*, 2016 ▸), where et_2_oen is *N*,*N*-di­ethyl­ethano­lamine. The columns are further linked together by Cu3 to form a structure with channels, into which projects the coordinated Cumeen_2_ unit, Fig. 3 ▸. The topology around Cu3 involves three 20-membered rings. There are four close inter­actions between amine N—H bonds and bridging CN groups, with N⋯N distances ranging from 3.257 (3) to 3.479 (3) Å, which may account for the tendency for ordered CN groups in this structure. The shortest H⋯H inter­molecular contact in (I) is 2.47 Å for H15*B⋯*H18*B*(−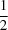 − *x*, −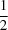 + *y*, −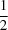 + *z*).

**Figure 5:**
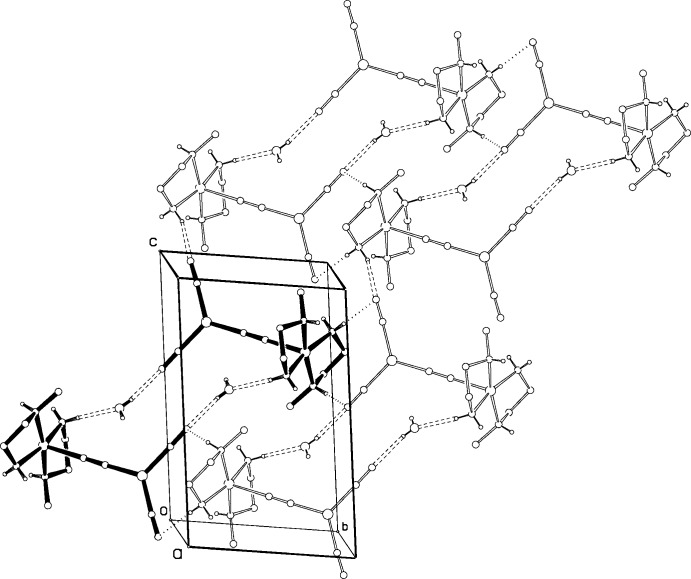
Packing diagram for compound (II), Cu_2_(CN)_3_meen_2_·H_2_O, looking approximately down the *a* axis. A hydrogen-bonded dimer is bolded, with hydrogen bonds shown as double-dashed lines. Hydrogen bonds linking dimers along the [011] direction are similarly shown. Possible attractive inter­actions between N—H groups and CN groups that would link dimers along the [110] direction are shown as single dashed lines.

Centrosymmetric pairs of discrete mol­ecules of (II) are held together by hydrogen bonding (Table 3 ▸) to the water mol­ecules, Fig. 5 ▸, with each water mol­ecule forming one donor and one acceptor hydrogen bond. These pairs are linked into chains *via* hydrogen bonds along [011], N4—H4*A*⋯⋯N3(1 − *x*, 1 − *y*, 2 − *z*)—C2(1 − *x*, 1 − *y*, 2 − *z*), where these four atoms are almost co-linear. Two other potentially attractive relationships between N—H bonds and cyanide groups are also shown in Fig. 5 ▸.

## Database survey   

Searches of the Cambridge Structure Database (CSD, Version 5.35; Groom *et al.*, 2016 ▸) yielded 53 structures containing the *M*meen fragment, where *M* is any metal. For 19 of these structures *M* = Cu (the Cumeen set) and for 19 *M* = Co (the Comeen set). There were four where *M* was a different metal, and 11 which involved duplicates or structures with no coordinates. The Cumeen set entries contained 24 chelate Cumeen rings, while the Comeen set contains 35 chelate rings.

The Cumeen set showed the same lengthening of the Cu—NH(CH_3_) bonds with respect to the Cu—NH_2_ bond lengths as found here in (I), with averages of 2.010 (4) and 2.041 (4) Å, respectively. A similar difference was found for the Comeen set, where the corresponding means were 1.962 (8) and 1.998 (8) Å. Cu—N bond lengths showed no correlation with coordination numbers around Cu, which ranged from four through six. N—Cu—N angles in the Cumeen set are in a limited range of 84.0 to 86.4°, and the four such angles in the present study all lie near the middle of this range.

The average of the absolute values of the N—C—C—N torsion angles in the chelate rings for the Cumeen set is 51.6°, with a sample s.u. of 6.5°, excluding one outlier from a flat chelate ring. Corresponding angles in the present work are all within one s.u. of the mean. The mean absolute CH_3_—N—C—C angles for *R*δ/*S*λ and *R*λ/*S*δ combinations, respectively, in the Cumeen set are 171 (6) and 89 (5)°, where sample s.u.’s are given. Equivalent torsion angles in both structures presented here fall within one s.u. of these means.

## Synthesis and crystallization   

The compounds were synthesized by air oxidation of CuCN/NaCN/meen systems. A typical preparation of (II) had CuCN (5.7 mmol) and NaCN (8.3 mmol) stirred in 6 mL of water until all solids dissolved, when 8.6 mmol of *N*-methyl­ethylenedi­amine (meen) in approximately 5 mL of water were added. Blue crystals in the form of extended thick plates were recovered after two days at room temperature. Crystals of (I) were obtained in a similar preparation with 11.5 mmmol CuCN, 16.5 mmol NaCN, and 16.2 mmol meen, to which were added an aqueous solution containing 9.9 mmol NaClO_4_. Blue crystals of (II) were obtained after two weeks, but after another six weeks the filtrate yielded large black polyhedral crystals of (I).

Infra-red spectra obtained with a Nicolet iS50 FT–IR machine on the polymer (I) showed three bands in the CN stretching region, with peaks at 2079, 2109, and 2119 cm^−1^. In addition, there are strong bands at 3250 and 3312 cm^−1^, and a weak, sharp band at 3150 cm^−1^, presumably all due to N-H stretching vibrations. For (II), CN stretching frequencies at 2089 and 2115 cm^−1^ were observed.

## Refinement details   

Crystal data, data collection and structure refinement details are summarized in Table 4 ▸. In (I), it was apparent that several low-order reflections were partially or completely obscured by the backstop and/or subject to overload. We recollected a fast dataset to θ = 15° with the backstop pushed back, obtained the scale factor between the two datasets using reflections with θ above 5°, and replaced 27 low-angle reflections in (I) with data from the fast dataset. Three low-angle reflections were not obtained in the fast dataset, and these have been omitted in the final refinement.

In (I), μ_3_-CN cyanide groups C1≡N1 and C2≡N2 were found to be ordered, with the C atom bridging two Cu atoms, as in Corfield *et al.* (2016 ▸). In addition, C5≡N5 was found to have a clearly preferred orientation and was refined as an ordered group. C,N occupancy factors were refined for the two other cyanide groups, with preferential occupancies of 79 (3)% and 78 (3)% found for C3≡N3 and C4≡N4, respectively. Only the major C or N atoms are listed in the cif tables of bond lengths and bond angles. In (II), all the CN groups are ordered; CN orientations were checked by refinements with inter­change of each CN group in turn, in each case resulting in significantly higher *R* factors.

In both compounds, C-bound H atoms were constrained to idealized positions with C—H distances of 0.97 Å for CH_2_ groups and 0.96 Å for CH_3_ groups, and *U*_eq_ values fixed at 1.2 times the *U*_iso_ of their bonded C atoms. The methyl torsion angles were refined. In (II), the N- and O-bound hydrogen atoms were clearly visible in the difference-Fourier map and were refined independently. The N-bound hydrogen atoms in (I) were clearly seen in a near-final difference map, and could be independently refined, but we chose to constrain them to idealized positions, with N—H distances of 0.90 Å for NH_2_ groups, 0.91 Å for NH groups, and *U*_eq_ values treated the same as for the C—H atoms.

For both (I) and (II), data had been previously collected with a CAD-4 system (Enraf–Nonius, 1994 ▸), on three crystals in the case of (I), and two crystals for (II). For (I), final *R*_1_ factors for the CAD-4 data were 0.045 for 2228 data with *F* ^2^ > 2σ, while for (II), *R*_1_ was 0.036 for 2245 data with *F* ^2^ > 2σ. It was felt instructive to compare refined parameters obtained by the two methods. We defined Δ/σ for a given parameter as the absolute value of the difference between the parameters determined by the two instruments divided by the square root of the sum of the squares of the standard deviations for the two parameters. For (I), the structural parameters agreed very well, for the mean and maximum Δ/σ for all parameters were 0.74 and 2.60. The maximum deviation for bond lengths was 2.1σ. For (II), there were differences of 4–5σ between positional parameters for the water oxygen atom, O1, which was much better defined in the data set from the KappaCCD instrument. Apart from parameters for O1, the agreement was excellent, with average Δ/σ for positional parameters 0.79, and no Δ/σ greater than 3. There were differences in the *U_ij_* for the two Cu atoms because an extinction parameter was refined for the KappaCCD data set. For all other atoms, the mean Δ/σ for the thermal parameters was 0.83 with only one Δ/σ greater than 3.

## Supplementary Material

Crystal structure: contains datablock(s) I, II. DOI: 10.1107/S2056989017000111/pk2594sup1.cif

Structure factors: contains datablock(s) I. DOI: 10.1107/S2056989017000111/pk2594Isup2.hkl

Structure factors: contains datablock(s) II. DOI: 10.1107/S2056989017000111/pk2594IIsup3.hkl

CCDC references: 1525492, 1525491

Additional supporting information:  crystallographic information; 3D view; checkCIF report

## Figures and Tables

**Table 1:** Selected bond lengths (Å) for (I)

Cu1—C1	1.980 (3)	Cu3—N2	1.942 (3)
Cu1—C2	2.042 (3)	Cu3—N3^ii^	1.961 (3)
Cu1—N4	2.050 (3)	Cu3—C5^iii^	1.910 (3)
Cu1—N5	2.041 (3)	Cu4—N11	2.002 (3)
Cu1—Cu2	2.5599 (7)	Cu4—N14	2.072 (3)
Cu2—C1	2.379 (3)	Cu4—N16	2.009 (3)
Cu2—C2	2.255 (3)	Cu4—N19	2.059 (3)
Cu2—C3	1.935 (3)	Cu4—N1	2.292 (3)
Cu2—C4^i^	1.948 (3)		

Symmetry codes: (i) 
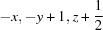
; (ii) 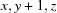; (iii) 
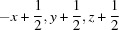
.

**Table 2:** Selected bond lengths (Å) for (II)

Cu1—C1	1.9434 (15)	Cu2—N4	2.0200 (14)
Cu1—C2	1.9380 (18)	Cu2—N7	2.0453 (13)
Cu1—C3	1.9414 (18)	Cu2—N14	2.0262 (15)
Cu2—N1	2.2232 (14)	Cu2—N17	2.0417 (14)

**Table 3:** Hydrogen-bond geometry (Å, °) for (II)

*D*—H⋯*A*	*D*—H	H⋯*A*	*D*⋯*A*	*D*—H⋯*A*
O1—H1⋯N2	0.68 (3)	2.13 (3)	2.810 (3)	171 (3)
N14—H14*A*⋯O1^i^	0.83 (2)	2.14 (2)	2.965 (3)	177 (2)
N4—H4*A*⋯N3^ii^	0.86 (2)	2.26 (2)	3.094 (2)	161.1 (19)
N7—H7⋯N2^iii^	0.810 (19)	2.460 (19)	3.162 (2)	145.7 (16)
N4—H4*B*⋯N3^iii^	0.78 (2)	2.53 (2)	3.302 (3)	170 (2)

Symmetry codes: (i) 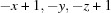; (ii) 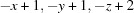; (iii) 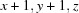.

**Table 4:** Experimental details

	(I)	(II)
Crystal data		
Chemical formula	[Cu_4_(CN)_5_(C_3_H_10_N_2_)_2_]	[Cu_2_(CN)_3_(C_3_H_10_N_2_)_2_]·H_2_O
*M*_r_	532.52	371.42
Crystal system, space group	Orthorhombic, *P* *n* *a*2_1_	Triclinic, *P* 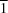
Temperature (K)	303	300
*a*, *b*, *c* (Å)	19.509 (2), 8.2306 (13), 11.100 (2)	7.5621 (2), 8.8689 (2), 12.8098 (3)
α, β, γ (°)	90, 90, 90	94.6851 (14), 101.8607 (12), 108.3780 (13)
*V* (Å^3^)	1782.3 (5)	787.91 (3)
*Z*	4	2
Radiation type	Mo *K*α	Mo *K*α
μ (mm^−1^)	4.72	2.70
Crystal size (mm)	0.5 × 0.4 × 0.4	0.5 × 0.4 × 0.3

Data collection		
Diffractometer	Enraf–Nonius KappaCCD	Enraf–Nonius KappaCCD
Absorption correction	Part of the refinement model (Δ*F*) (*SCALEPACK*; Otwinowski & Minor, 1997 ▸)	Part of the refinement model (Δ*F*) (*SCALEPACK*; Otwinowski & Minor, 1997 ▸)
*T*_min_, *T*_max_	0.103, 0.146	0.31, 0.39
No. of measured, independent and observed [*I* > 2σ(*I*)] reflections	15054, 4062, 3964	24810, 3611, 3387
*R*_int_	0.037	0.029
(sin θ/λ)_max_ (Å^−1^)	0.649	0.650

Refinement		
*R*[*F* ^2^ > 2σ(*F* ^2^)], *wR*(*F* ^2^), *S*	0.018, 0.045, 1.05	0.021, 0.053, 1.07
No. of reflections	4062	3611
No. of parameters	221	207
No. of restraints	1	0
H-atom treatment	H-atom parameters constrained	H atoms treated by a mixture of independent and constrained refinement
Δρ_max_, Δρ_min_ (e Å^−3^)	0.25, −0.38	0.31, −0.26
Absolute structure	Flack *x* determined using 1806 quotients [(*I* ^+^)−(*I* ^−^)]/[(*I* ^+^)+(*I* ^−^)] (Parsons *et al.*, 2013 ▸)	–
Absolute structure parameter	0.010 (9)	–

Computer programs: *KappaCCD Server Software* (Nonius, 1997 ▸), *DENZO* and *SCALEPACK* (Otwinowski & Minor, 1997 ▸), *SHELXS97* (Sheldrick, 2008 ▸), *SHELXL2014* (Sheldrick, 2015 ▸), *ORTEPIII* (Burnett & Johnson, 1996 ▸) and *publCIF* (Westrip, 2010 ▸).

## supplementary crystallographic information

### (I) Poly[bis(µ3-cyanido-κ3C:C:N)tris(µ2-cyanido-κ2C:N)bis(N-methylethane-1,2-diamine\- κ2N,N')tricopper(I)copper(II)] Crystal data

**Table:**

[Cu_4_(CN)_5_(C_3_H_10_N_2_)_2_]	*F*(000) = 1060
*M**_r_* = 532.52	*D*_x_ = 1.985 Mg m^−^^3^*D*_m_ = 2.01 (7) Mg m^−^^3^*D*_m_ measured by flotation in 1,2-dibromopropane/1,2,3-trichloropropane mixtures. Three independent determinations were made.
Orthorhombic, *P**n**a*2_1_	Mo *K*α radiation, λ = 0.71070 Å
Hall symbol: P 2c -2n	Cell parameters from 474 reflections
*a* = 19.509 (2) Å	θ = 1.8–20.2°
*b* = 8.2306 (13) Å	µ = 4.72 mm^−^^1^
*c* = 11.100 (2) Å	*T* = 303 K
*V* = 1782.3 (5) Å^3^	Block cut from large polyhedral crystal, black
*Z* = 4	0.5 × 0.4 × 0.4 mm

### (I) Poly[bis(µ3-cyanido-κ3C:C:N)tris(µ2-cyanido-κ2C:N)bis(N-methylethane-1,2-diamine\- κ2N,N')tricopper(I)copper(II)] Data collection

**Table:**

Enraf–Nonius KappaCCD diffractometer	4062 independent reflections
Radiation source: fine-focus sealed tube	3964 reflections with *I* > 2σ(*I*)
Graphite monochromator	*R*_int_ = 0.037
Detector resolution: 9 pixels mm^-1^	θ_max_ = 27.5°, θ_min_ = 2.1°
combination of ω and φ scans	*h* = 0→25
Absorption correction: part of the refinement model (Δ*F*) (SCALEPACK; Otwinowski & Minor, 1997)	*k* = 0→10
*T*_min_ = 0.103, *T*_max_ = 0.146	*l* = −14→14
15054 measured reflections	

### (I) Poly[bis(µ3-cyanido-κ3C:C:N)tris(µ2-cyanido-κ2C:N)bis(N-methylethane-1,2-diamine\- κ2N,N')tricopper(I)copper(II)] Refinement

**Table:**

Refinement on *F*^2^	Secondary atom site location: difference Fourier map
Least-squares matrix: full	Hydrogen site location: inferred from neighbouring sites
*R*[*F*^2^ > 2σ(*F*^2^)] = 0.018	H-atom parameters constrained
*wR*(*F*^2^) = 0.045	*w* = 1/[σ^2^(*F*_o_^2^) + (0.022*P*)^2^ + 0.040*P*] where *P* = (*F*_o_^2^ + 2*F*_c_^2^)/3
*S* = 1.05	(Δ/σ)_max_ < 0.001
4062 reflections	Δρ_max_ = 0.25 e Å^−^^3^
221 parameters	Δρ_min_ = −0.38 e Å^−^^3^
1 restraint	Absolute structure: Flack *x* determined using 1806 quotients [(*I*^+^)-(*I*^-^)]/[(*I*^+^)+(*I*^-^)] (Parsons *et al.*, 2013)
Primary atom site location: structure-invariant direct methods	Absolute structure parameter: 0.010 (9)

### (I) Poly[bis(µ3-cyanido-κ3C:C:N)tris(µ2-cyanido-κ2C:N)bis(N-methylethane-1,2-diamine\- κ2N,N')tricopper(I)copper(II)] Special details

**Table:**

Experimental. Scalepack values for Tmin and Tmax are normalized to unity. Values given here were obtained by multiplying them by exp(-µd) where d= 0.45mm.
Geometry. All esds (except the esd in the dihedral angle between two l.s. planes) are estimated using the full covariance matrix. The cell esds are taken into account individually in the estimation of esds in distances, angles and torsion angles; correlations between esds in cell parameters are only used when they are defined by crystal symmetry. An approximate (isotropic) treatment of cell esds is used for estimating esds involving l.s. planes.
Refinement. Refinement of F^2^ against ALL reflections. The weighted R-factor wR and goodness of fit S are based on F^2^, conventional R-factors R are based on F, with F set to zero for negative F^2^. The threshold expression of F^2^ > 2sigma(F^2^) is used only for calculating R-factors(gt) etc. and is not relevant to the choice of reflections for refinement. R-factors based on F^2^ are statistically about twice as large as those based on F, and R- factors based on ALL data will be even larger.

### (I) Poly[bis(µ3-cyanido-κ3C:C:N)tris(µ2-cyanido-κ2C:N)bis(N-methylethane-1,2-diamine\- κ2N,N')tricopper(I)copper(II)] Fractional atomic coordinates and isotropic or equivalent isotropic displacement parameters (Å^2^)

**Table:**

	*x*	*y*	*z*	*U*_iso_*/*U*_eq_	Occ. (<1)
Cu1	0.07836 (2)	0.38153 (5)	0.00021 (3)	0.02552 (9)	
Cu2	0.06847 (2)	0.28058 (5)	0.21765 (4)	0.03143 (10)	
Cu3	0.20761 (2)	0.76738 (5)	0.26057 (4)	0.03009 (10)	
Cu4	−0.11518 (2)	−0.01338 (4)	0.15874 (4)	0.02352 (9)	
C1	0.00592 (15)	0.2214 (4)	0.0390 (3)	0.0252 (6)	
N1	−0.03914 (15)	0.1363 (3)	0.0471 (3)	0.0358 (7)	
C2	0.12936 (15)	0.4885 (4)	0.1399 (3)	0.0279 (7)	
N2	0.16340 (14)	0.5829 (3)	0.1840 (2)	0.0318 (6)	
C3	0.12207 (16)	0.0852 (4)	0.2405 (3)	0.0326 (9)	0.79 (3)
N3	0.15237 (15)	−0.0341 (4)	0.2510 (3)	0.0402 (9)	0.79 (3)
N3A	0.12207 (16)	0.0852 (4)	0.2405 (3)	0.0326 (9)	0.21 (3)
C3A	0.15237 (15)	−0.0341 (4)	0.2510 (3)	0.0402 (9)	0.21 (3)
C4	−0.00363 (15)	0.6193 (4)	−0.1714 (3)	0.0270 (8)	0.78 (3)
N4	0.03183 (14)	0.5501 (4)	−0.1084 (3)	0.0298 (7)	0.78 (3)
N4A	−0.00363 (15)	0.6193 (4)	−0.1714 (3)	0.0270 (8)	0.22 (3)
C4A	0.03183 (14)	0.5501 (4)	−0.1084 (3)	0.0298 (7)	0.22 (3)
C5	0.20639 (16)	0.2799 (4)	−0.1578 (3)	0.0274 (7)	
N5	0.15765 (14)	0.3019 (4)	−0.1041 (3)	0.0311 (6)	
N11	−0.03685 (14)	−0.1076 (3)	0.2519 (3)	0.0332 (6)	
H11A	−0.0058	−0.0310	0.2667	0.040*	
H11B	−0.0518	−0.1462	0.3221	0.040*	
C12	−0.00592 (18)	−0.2392 (4)	0.1810 (4)	0.0410 (9)	
H12A	0.0220	−0.3080	0.2323	0.049*	
H12B	0.0230	−0.1946	0.1181	0.049*	
C13	−0.06375 (19)	−0.3366 (5)	0.1261 (3)	0.0385 (8)	
H13A	−0.0455	−0.4207	0.0740	0.046*	
H13B	−0.0906	−0.3880	0.1890	0.046*	
N14	−0.10737 (15)	−0.2222 (3)	0.0553 (3)	0.0310 (6)	
H14	−0.0807	−0.1925	−0.0161	0.037*	
C15	−0.1702 (2)	−0.3019 (5)	0.0107 (4)	0.0465 (9)	
H15A	−0.1581	−0.3981	−0.0333	0.056*	
H15B	−0.1945	−0.2286	−0.0413	0.056*	
H15C	−0.1988	−0.3307	0.0777	0.056*	
N16	−0.19542 (14)	0.0806 (4)	0.0694 (3)	0.0329 (6)	
H16A	−0.1875	0.0790	−0.0096	0.040*	
H16B	−0.2331	0.0230	0.0843	0.040*	
C17	−0.2044 (2)	0.2508 (5)	0.1118 (4)	0.0470 (10)	
H17A	−0.2484	0.2933	0.0863	0.056*	
H17B	−0.1685	0.3199	0.0797	0.056*	
C18	−0.2003 (2)	0.2437 (5)	0.2477 (4)	0.0479 (10)	
H18A	−0.2019	0.3528	0.2805	0.058*	
H18B	−0.2392	0.1835	0.2790	0.058*	
N19	−0.13606 (15)	0.1637 (3)	0.2845 (3)	0.0326 (6)	
H19	−0.1000	0.2456	0.2755	0.039*	
C20	−0.1379 (2)	0.1228 (5)	0.4134 (4)	0.0493 (10)	
H20A	−0.1495	0.2180	0.4591	0.059*	
H20B	−0.0938	0.0837	0.4381	0.059*	
H20C	−0.1717	0.0401	0.4270	0.059*	

### (I) Poly[bis(µ3-cyanido-κ3C:C:N)tris(µ2-cyanido-κ2C:N)bis(N-methylethane-1,2-diamine\- κ2N,N')tricopper(I)copper(II)] Atomic displacement parameters (Å^2^)

**Table:**

	*U*^11^	*U*^22^	*U*^33^	*U*^12^	*U*^13^	*U*^23^
Cu1	0.02310 (16)	0.02762 (19)	0.02583 (18)	−0.00503 (14)	0.00102 (15)	−0.00028 (16)
Cu2	0.0336 (2)	0.0315 (2)	0.0292 (2)	0.00564 (16)	0.00206 (18)	−0.00226 (18)
Cu3	0.0263 (2)	0.0298 (2)	0.0342 (2)	−0.00366 (15)	−0.00764 (16)	−0.00447 (18)
Cu4	0.02156 (16)	0.02424 (18)	0.02476 (16)	−0.00003 (14)	−0.00319 (14)	0.00006 (16)
C1	0.0245 (14)	0.0257 (15)	0.0254 (14)	−0.0005 (12)	0.0016 (12)	−0.0003 (12)
N1	0.0315 (15)	0.0403 (17)	0.0357 (15)	−0.0123 (13)	−0.0017 (12)	0.0058 (14)
C2	0.0212 (12)	0.0253 (15)	0.037 (2)	0.0006 (11)	−0.0041 (14)	−0.0099 (14)
N2	0.0294 (14)	0.0266 (13)	0.0393 (17)	0.0029 (12)	−0.0061 (12)	−0.0057 (12)
C3	0.0304 (16)	0.0313 (19)	0.0362 (18)	−0.0003 (13)	−0.0063 (14)	−0.0003 (14)
N3	0.0344 (16)	0.0287 (17)	0.057 (2)	0.0015 (13)	−0.0138 (16)	−0.0037 (15)
N3A	0.0304 (16)	0.0313 (19)	0.0362 (18)	−0.0003 (13)	−0.0063 (14)	−0.0003 (14)
C3A	0.0344 (16)	0.0287 (17)	0.057 (2)	0.0015 (13)	−0.0138 (16)	−0.0037 (15)
C4	0.0283 (15)	0.0289 (16)	0.0238 (15)	−0.0012 (12)	0.0002 (13)	−0.0014 (13)
N4	0.0314 (15)	0.0315 (15)	0.0266 (14)	−0.0005 (13)	0.0004 (13)	0.0030 (12)
N4A	0.0283 (15)	0.0289 (16)	0.0238 (15)	−0.0012 (12)	0.0002 (13)	−0.0014 (13)
C4A	0.0314 (15)	0.0315 (15)	0.0266 (14)	−0.0005 (13)	0.0004 (13)	0.0030 (12)
C5	0.0253 (15)	0.0267 (16)	0.0302 (16)	−0.0017 (12)	0.0048 (13)	−0.0010 (13)
N5	0.0243 (13)	0.0395 (16)	0.0294 (14)	0.0022 (12)	0.0009 (12)	−0.0018 (12)
N11	0.0285 (14)	0.0406 (16)	0.0304 (14)	−0.0012 (11)	−0.0032 (12)	0.0102 (13)
C12	0.0286 (17)	0.044 (2)	0.050 (2)	0.0134 (15)	0.0098 (15)	0.0161 (17)
C13	0.0449 (19)	0.0286 (17)	0.042 (2)	0.0111 (15)	0.0160 (16)	0.0038 (16)
N14	0.0369 (15)	0.0304 (14)	0.0256 (13)	−0.0031 (12)	0.0052 (12)	0.0006 (12)
C15	0.056 (2)	0.044 (2)	0.040 (2)	−0.0105 (19)	0.000 (2)	−0.0112 (18)
N16	0.0282 (13)	0.0346 (15)	0.0361 (15)	−0.0009 (12)	−0.0053 (12)	−0.0008 (13)
C17	0.048 (2)	0.034 (2)	0.059 (3)	0.0104 (17)	−0.010 (2)	0.0016 (18)
C18	0.039 (2)	0.042 (2)	0.063 (3)	0.0087 (16)	−0.002 (2)	−0.011 (2)
N19	0.0332 (14)	0.0287 (14)	0.0360 (16)	−0.0042 (12)	−0.0013 (12)	−0.0056 (12)
C20	0.066 (3)	0.048 (2)	0.0340 (19)	−0.005 (2)	0.0050 (19)	−0.0140 (17)

### (I) Poly[bis(µ3-cyanido-κ3C:C:N)tris(µ2-cyanido-κ2C:N)bis(N-methylethane-1,2-diamine\- κ2N,N')tricopper(I)copper(II)] Geometric parameters (Å, º)

**Table:**

Cu1—C1	1.980 (3)	C12—C13	1.512 (6)
Cu1—C2	2.042 (3)	C12—H12A	0.9700
Cu1—N4	2.050 (3)	C12—H12B	0.9700
Cu1—N5	2.041 (3)	C13—N14	1.493 (5)
Cu1—Cu2	2.5599 (7)	C13—H13A	0.9700
Cu2—C1	2.379 (3)	C13—H13B	0.9700
Cu2—C2	2.255 (3)	N14—C15	1.475 (5)
Cu2—C3	1.935 (3)	N14—H14	0.9800
Cu2—C4^i^	1.948 (3)	C15—H15A	0.9600
Cu3—N2	1.942 (3)	C15—H15B	0.9600
Cu3—N3^ii^	1.961 (3)	C15—H15C	0.9600
Cu3—C5^iii^	1.910 (3)	N16—C17	1.488 (5)
Cu4—N11	2.002 (3)	N16—H16A	0.8900
Cu4—N14	2.072 (3)	N16—H16B	0.8900
Cu4—N16	2.009 (3)	C17—C18	1.512 (7)
Cu4—N19	2.059 (3)	C17—H17A	0.9700
Cu4—N1	2.292 (3)	C17—H17B	0.9700
C1—N1	1.127 (4)	C18—N19	1.474 (5)
C2—N2	1.133 (4)	C18—H18A	0.9700
C3—N3	1.152 (4)	C18—H18B	0.9700
C4—N4	1.137 (4)	N19—C20	1.470 (5)
C5—N5	1.136 (4)	N19—H19	0.9800
N11—C12	1.469 (5)	C20—H20A	0.9600
N11—H11A	0.8900	C20—H20B	0.9600
N11—H11B	0.8900	C20—H20C	0.9600
			
C1—Cu1—C2	118.01 (13)	N11—C12—H12A	110.2
C1—Cu1—N4	105.22 (12)	C13—C12—H12A	110.2
C1—Cu1—N5	116.79 (13)	N11—C12—H12B	110.2
C2—Cu1—N4	111.75 (12)	C13—C12—H12B	110.2
C2—Cu1—N5	101.54 (12)	H12A—C12—H12B	108.5
N4—Cu1—N5	102.67 (12)	C12—C13—N14	107.6 (3)
C1—Cu1—Cu2	61.64 (10)	C12—C13—H13A	110.2
C2—Cu1—Cu2	57.36 (9)	C12—C13—H13B	110.2
N4—Cu1—Cu2	137.79 (8)	N14—C13—H13A	110.2
N5—Cu1—Cu2	119.22 (8)	N14—C13—H13B	110.2
C1—Cu2—C2	96.13 (11)	H13A—C13—H13B	108.5
C1—Cu2—C3	102.49 (12)	C13—N14—C15	111.7 (3)
C1—Cu2—C4^i^	106.29 (12)	C13—N14—Cu4	105.9 (2)
C2—Cu2—C3	113.35 (12)	C15—N14—Cu4	119.6 (2)
C2—Cu2—C4^i^	105.25 (12)	C13—N14—H14	106.3
C3—Cu2—C4^i^	128.27 (13)	C15—N14—H14	106.3
C1—Cu2—Cu1	47.10 (7)	Cu4—N14—H14	106.3
C2—Cu2—Cu1	49.69 (8)	N14—C15—H15A	109.5
C3—Cu2—Cu1	110.66 (10)	N14—C15—H15B	109.5
C4^i^—Cu2—Cu1	120.51 (9)	N14—C15—H15C	109.5
N2—Cu3—C5^iii^	129.79 (13)	H15A—C15—H15B	109.5
N2—Cu3—N3^ii^	112.56 (12)	H15A—C15—H15C	109.5
N3^ii^—Cu3—C5^iii^	117.64 (13)	H15B—C15—H15C	109.5
N11—Cu4—N14	84.76 (12)	C17—N16—Cu4	107.3 (2)
N16—Cu4—N19	84.72 (12)	C17—N16—H16A	110.2
N11—Cu4—N16	178.39 (13)	C17—N16—H16B	110.2
N14—Cu4—N19	167.74 (11)	Cu4—N16—H16A	110.2
N11—Cu4—N19	94.30 (12)	Cu4—N16—H16B	110.2
N14—Cu4—N16	95.92 (12)	H16A—N16—H16B	108.5
N1—Cu4—N11	89.64 (11)	N16—C17—C18	105.8 (3)
N1—Cu4—N14	95.67 (11)	N16—C17—H17A	110.6
N1—Cu4—N16	91.74 (12)	N16—C17—H17B	110.6
N1—Cu4—N19	96.55 (11)	C18—C17—H17A	110.6
N1—C1—Cu1	170.9 (3)	C18—C17—H17B	110.6
N1—C1—Cu2	117.4 (3)	H17A—C17—H17B	108.7
Cu1—C1—Cu2	71.26 (10)	C17—C18—N19	109.8 (3)
C1—N1—Cu4	151.8 (3)	C17—C18—H18A	109.7
N2—C2—Cu1	155.4 (3)	C17—C18—H18B	109.7
N2—C2—Cu2	131.6 (3)	N19—C18—H18A	109.7
Cu1—C2—Cu2	72.94 (9)	N19—C18—H18B	109.7
C2—N2—Cu3	170.2 (3)	H18A—C18—H18B	108.2
N3—C3—Cu2	177.3 (3)	C18—N19—C20	110.6 (3)
C3—N3—Cu3^iv^	176.5 (3)	C18—N19—Cu4	107.2 (2)
N4—C4—Cu2^v^	174.9 (3)	C20—N19—Cu4	120.2 (2)
C4—N4—Cu1	166.0 (3)	Cu4—N19—H19	106.0
N5—C5—Cu3^vi^	172.9 (3)	C18—N19—H19	106.0
C5—N5—Cu1	169.4 (3)	C20—N19—H19	106.0
C12—N11—Cu4	108.8 (2)	N19—C20—H20A	109.5
Cu4—N11—H11A	109.9	N19—C20—H20B	109.5
Cu4—N11—H11B	109.9	N19—C20—H20C	109.5
C12—N11—H11A	109.9	H20A—C20—H20B	109.5
C12—N11—H11B	109.9	H20A—C20—H20C	109.5
H11A—N11—H11B	108.3	H20B—C20—H20C	109.5
N11—C12—C13	107.5 (3)		
			
N14—C13—C12—N11	56.0 (4)	C20—N19—C18—C17	−167.4 (3)
N19—C18—C17—N16	54.6 (4)	C15—N14—Cu4—N11	141.85 (19)
C15—N14—C13—C12	−172.8 (3)	C20—N19—Cu4—N16	133.9 (2)

Symmetry codes: (i) −*x*, −*y*+1, *z*+1/2; (ii) *x*, *y*+1, *z*; (iii) −*x*+1/2, *y*+1/2, *z*+1/2; (iv) *x*, *y*−1, *z*; (v) −*x*, −*y*+1, *z*−1/2; (vi) −*x*+1/2, *y*−1/2, *z*−1/2.

### (II) [(N-Methylethylenediamine-κ2N,N')copper(II)]-µ2-cyanido-κ2C:N-[bis(cyanido-κC)copper(I)] monohydrate Crystal data

**Table:**

[Cu_2_(CN)_3_(C_3_H_10_N_2_)_2_]·H_2_O	*Z* = 2
*M**_r_* = 371.42	*F*(000) = 382
Triclinic, *P*1	*D*_x_ = 1.566 Mg m^−^^3^*D*_m_ = 1.570 (5) Mg m^−^^3^*D*_m_ measured by flotation in 1,2-dibromopropane/1,2,3-trichloropropane mixtures. Four independent determinations were made.
Hall symbol: -P 1	Mo *K*α radiation, λ = 0.71070 Å
*a* = 7.5621 (2) Å	Cell parameters from 3501 reflections
*b* = 8.8689 (2) Å	θ = 1.8–27.5°
*c* = 12.8098 (3) Å	µ = 2.70 mm^−^^1^
α = 94.6851 (14)°	*T* = 300 K
β = 101.8607 (12)°	Block cut from large elongated plate, blue
γ = 108.3780 (13)°	0.5 × 0.4 × 0.3 mm
*V* = 787.91 (3) Å^3^	

### (II) [(N-Methylethylenediamine-κ2N,N')copper(II)]-µ2-cyanido-κ2C:N-[bis(cyanido-κC)copper(I)] monohydrate Data collection

**Table:**

Enraf–Nonius KappaCCD diffractometer	3611 independent reflections
Radiation source: fine-focus sealed tube	3387 reflections with *I* > 2σ(*I*)
Graphite monochromator	*R*_int_ = 0.029
Detector resolution: 9 pixels mm^-1^	θ_max_ = 27.5°, θ_min_ = 3.0°
combination of ω and φ scans	*h* = 0→9
Absorption correction: part of the refinement model (Δ*F*) (SCALEPACK; Otwinowski & Minor, 1997)	*k* = −11→10
*T*_min_ = 0.31, *T*_max_ = 0.39	*l* = −16→16
24810 measured reflections	

### (II) [(N-Methylethylenediamine-κ2N,N')copper(II)]-µ2-cyanido-κ2C:N-[bis(cyanido-κC)copper(I)] monohydrate Refinement

**Table:**

Refinement on *F*^2^	Primary atom site location: heavy-atom method
Least-squares matrix: full	Secondary atom site location: difference Fourier map
*R*[*F*^2^ > 2σ(*F*^2^)] = 0.021	Hydrogen site location: inferred from neighbouring sites
*wR*(*F*^2^) = 0.053	H atoms treated by a mixture of independent and constrained refinement
*S* = 1.07	*w* = 1/[σ^2^(*F*_o_^2^) + (0.023*P*)^2^ + 0.270*P*] where *P* = (*F*_o_^2^ + 2*F*_c_^2^)/3
3611 reflections	(Δ/σ)_max_ = 0.001
207 parameters	Δρ_max_ = 0.31 e Å^−^^3^
0 restraints	Δρ_min_ = −0.26 e Å^−^^3^

### (II) [(N-Methylethylenediamine-κ2N,N')copper(II)]-µ2-cyanido-κ2C:N-[bis(cyanido-κC)copper(I)] monohydrate Special details

**Table:**

Experimental. Scalepack values for Tmin and Tmax are normalized to unity. Values given here were obtained by multiplying them by exp(-µd) where d= crystal_size_mid.
Geometry. All esds (except the esd in the dihedral angle between two l.s. planes) are estimated using the full covariance matrix. The cell esds are taken into account individually in the estimation of esds in distances, angles and torsion angles; correlations between esds in cell parameters are only used when they are defined by crystal symmetry. An approximate (isotropic) treatment of cell esds is used for estimating esds involving l.s. planes.
Refinement. Refinement of F^2^ against ALL reflections. The weighted R-factor wR and goodness of fit S are based on F^2^, conventional R-factors R are based on F, with F set to zero for negative F^2^. The threshold expression of F^2^ > 2sigma(F^2^) is used only for calculating R-factors(gt) etc. and is not relevant to the choice of reflections for refinement. R-factors based on F^2^ are statistically about twice as large as those based on F, and R- factors based on ALL data will be even larger.

### (II) [(N-Methylethylenediamine-κ2N,N')copper(II)]-µ2-cyanido-κ2C:N-[bis(cyanido-κC)copper(I)] monohydrate Fractional atomic coordinates and isotropic or equivalent isotropic displacement parameters (Å^2^)

**Table:**

	*x*	*y*	*z*	*U*_iso_*/*U*_eq_	
Cu1	0.44394 (3)	0.21586 (2)	0.789560 (16)	0.04033 (7)	
Cu2	0.98671 (2)	0.744243 (19)	0.753969 (13)	0.03124 (7)	
O1	0.1896 (5)	−0.2985 (3)	0.4215 (2)	0.0992 (8)	
C1	0.6318 (2)	0.41700 (18)	0.77647 (12)	0.0379 (3)	
N1	0.7425 (2)	0.53316 (17)	0.76709 (12)	0.0461 (3)	
C2	0.3303 (3)	0.0522 (2)	0.66225 (15)	0.0488 (4)	
N2	0.2639 (3)	−0.0419 (2)	0.58678 (15)	0.0696 (5)	
C3	0.3714 (3)	0.1763 (2)	0.92430 (15)	0.0503 (4)	
N3	0.3261 (3)	0.1471 (2)	1.00140 (15)	0.0714 (5)	
N4	0.9197 (2)	0.91902 (18)	0.83087 (11)	0.0393 (3)	
H4A	0.841 (3)	0.877 (3)	0.8693 (18)	0.056 (6)*	
H4B	1.009 (3)	0.981 (3)	0.8724 (18)	0.057 (6)*	
C5	0.8289 (3)	0.9995 (2)	0.75082 (15)	0.0544 (4)	
H5A	0.9265	1.0869	0.7325	0.065*	
H5B	0.7443	1.0439	0.7804	0.065*	
C6	0.7160 (3)	0.8774 (2)	0.65180 (14)	0.0494 (4)	
H6A	0.6080	0.7976	0.6680	0.059*	
H6B	0.6668	0.9296	0.5947	0.059*	
N7	0.8438 (2)	0.79978 (16)	0.61706 (10)	0.0377 (3)	
H7	0.927 (3)	0.868 (2)	0.5989 (14)	0.036 (4)*	
C8	0.7415 (4)	0.6680 (3)	0.52599 (15)	0.0650 (6)	
H8A	0.6582	0.5794	0.5504	0.078*	
H8B	0.8330	0.6337	0.4976	0.078*	
H8C	0.6667	0.7049	0.4705	0.078*	
N14	1.1101 (3)	0.6146 (2)	0.67452 (13)	0.0494 (4)	
H14A	1.025 (3)	0.528 (3)	0.6453 (18)	0.058 (7)*	
H14B	1.150 (4)	0.660 (3)	0.631 (2)	0.070 (7)*	
C15	1.2645 (3)	0.5852 (3)	0.75204 (19)	0.0673 (6)	
H15A	1.3854	0.6713	0.7594	0.081*	
H15B	1.2785	0.4847	0.7262	0.081*	
C16	1.2143 (3)	0.5773 (2)	0.85903 (16)	0.0529 (4)	
H16A	1.1029	0.4828	0.8543	0.064*	
H16B	1.3208	0.5711	0.9134	0.064*	
N17	1.1722 (2)	0.72477 (17)	0.88834 (11)	0.0426 (3)	
H17	1.266 (3)	0.798 (3)	0.8945 (16)	0.047 (5)*	
C18	1.1031 (3)	0.7205 (2)	0.98757 (14)	0.0569 (5)	
H18A	0.9743	0.6456	0.9725	0.068*	
H18B	1.1048	0.8257	1.0135	0.068*	
H18C	1.1852	0.6873	1.0416	0.068*	
H1	0.214 (4)	−0.230 (3)	0.458 (2)	0.069 (8)*	
H2	0.253 (5)	−0.289 (4)	0.392 (3)	0.086 (13)*	

### (II) [(N-Methylethylenediamine-κ2N,N')copper(II)]-µ2-cyanido-κ2C:N-[bis(cyanido-κC)copper(I)] monohydrate Atomic displacement parameters (Å^2^)

**Table:**

	*U*^11^	*U*^22^	*U*^33^	*U*^12^	*U*^13^	*U*^23^
Cu1	0.03480 (11)	0.03580 (11)	0.04541 (12)	0.00351 (8)	0.01158 (8)	0.00885 (8)
Cu2	0.03185 (10)	0.02916 (10)	0.03047 (10)	0.00749 (7)	0.00698 (7)	0.00575 (7)
O1	0.136 (2)	0.0550 (11)	0.1022 (17)	0.0208 (12)	0.0481 (16)	−0.0130 (11)
C1	0.0331 (7)	0.0377 (8)	0.0382 (8)	0.0066 (6)	0.0062 (6)	0.0089 (6)
N1	0.0375 (7)	0.0418 (7)	0.0521 (8)	0.0031 (6)	0.0099 (6)	0.0152 (6)
C2	0.0481 (9)	0.0403 (8)	0.0518 (10)	0.0015 (7)	0.0205 (8)	0.0076 (7)
N2	0.0754 (12)	0.0553 (10)	0.0621 (11)	−0.0037 (9)	0.0292 (9)	−0.0063 (8)
C3	0.0435 (9)	0.0453 (9)	0.0498 (10)	−0.0028 (7)	0.0145 (7)	0.0035 (7)
N3	0.0725 (12)	0.0741 (12)	0.0616 (11)	0.0064 (9)	0.0326 (9)	0.0116 (9)
N4	0.0424 (7)	0.0391 (7)	0.0358 (7)	0.0129 (6)	0.0104 (6)	0.0043 (6)
C5	0.0739 (12)	0.0538 (10)	0.0511 (10)	0.0395 (10)	0.0190 (9)	0.0134 (8)
C6	0.0477 (9)	0.0651 (11)	0.0446 (9)	0.0282 (8)	0.0129 (7)	0.0201 (8)
N7	0.0416 (7)	0.0374 (7)	0.0330 (6)	0.0101 (6)	0.0105 (5)	0.0103 (5)
C8	0.0863 (15)	0.0552 (11)	0.0399 (10)	0.0241 (10)	−0.0112 (9)	0.0005 (8)
N14	0.0605 (10)	0.0555 (10)	0.0452 (8)	0.0287 (8)	0.0241 (8)	0.0142 (7)
C15	0.0586 (12)	0.0890 (16)	0.0741 (14)	0.0441 (12)	0.0261 (10)	0.0202 (12)
C16	0.0479 (10)	0.0563 (10)	0.0569 (11)	0.0258 (8)	0.0038 (8)	0.0130 (8)
N17	0.0384 (7)	0.0382 (7)	0.0421 (7)	0.0073 (6)	−0.0003 (6)	0.0046 (6)
C18	0.0737 (13)	0.0592 (11)	0.0359 (9)	0.0262 (10)	0.0025 (8)	0.0113 (8)

### (II) [(N-Methylethylenediamine-κ2N,N')copper(II)]-µ2-cyanido-κ2C:N-[bis(cyanido-κC)copper(I)] monohydrate Geometric parameters (Å, º)

**Table:**

Cu1—C1	1.9434 (15)	C6—H6B	0.9700
Cu1—C2	1.9380 (18)	N7—C8	1.470 (2)
Cu1—C3	1.9414 (18)	N7—H7	0.810 (19)
Cu2—N1	2.2232 (14)	C8—H8A	0.9600
Cu2—N4	2.0200 (14)	C8—H8B	0.9600
Cu2—N7	2.0453 (13)	C8—H8C	0.9600
Cu2—N14	2.0262 (15)	N14—C15	1.475 (3)
Cu2—N17	2.0417 (14)	N14—H14A	0.83 (2)
O1—H1	0.68 (3)	N14—H14B	0.77 (3)
O1—H2	0.66 (3)	C15—C16	1.497 (3)
C1—N1	1.138 (2)	C15—H15A	0.9700
C2—N2	1.135 (2)	C15—H15B	0.9700
C3—N3	1.134 (2)	C16—N17	1.478 (2)
N4—C5	1.474 (2)	C16—H16A	0.9700
N4—H4A	0.86 (2)	C16—H16B	0.9700
N4—H4B	0.78 (2)	N17—C18	1.469 (2)
C5—C6	1.503 (3)	N17—H17	0.78 (2)
C5—H5A	0.9700	C18—H18A	0.9600
C5—H5B	0.9700	C18—H18B	0.9600
C6—N7	1.467 (2)	C18—H18C	0.9600
C6—H6A	0.9700		
			
C1—Cu1—C2	117.49 (7)	C6—N7—C8	112.25 (16)
C1—Cu1—C3	122.15 (7)	C6—N7—Cu2	105.83 (10)
C2—Cu1—C3	120.36 (7)	C8—N7—Cu2	117.11 (11)
N1—Cu2—N4	98.67 (6)	C6—N7—H7	107.8 (13)
N1—Cu2—N14	95.33 (7)	C8—N7—H7	109.2 (13)
N1—Cu2—N17	94.80 (6)	Cu2—N7—H7	104.0 (13)
N1—Cu2—N7	96.00 (6)	N7—C8—H8A	109.5
N4—Cu2—N7	84.33 (6)	N7—C8—H8B	109.5
N14—Cu2—N17	84.09 (6)	H8A—C8—H8B	109.5
N4—Cu2—N14	165.99 (7)	N7—C8—H8C	109.5
N7—Cu2—N17	169.19 (6)	H8A—C8—H8C	109.5
N4—Cu2—N17	94.23 (6)	H8B—C8—H8C	109.5
N7—Cu2—N14	94.71 (6)	C15—N14—Cu2	109.64 (13)
H1—O1—H2	109 (4)	C15—N14—H14A	110.0 (16)
N1—C1—Cu1	178.61 (16)	Cu2—N14—H14A	107.1 (16)
C1—N1—Cu2	172.56 (14)	C15—N14—H14B	110.8 (19)
N2—C2—Cu1	178.96 (18)	Cu2—N14—H14B	109.6 (19)
C2—N2—H1	163.7 (8)	H14A—N14—H14B	110 (2)
C2—N2—H7^i^	99.5 (4)	N14—C15—C16	108.87 (15)
H1—N2—H7^i^	93.6 (8)	N14—C15—H15A	109.9
N3—C3—Cu1	177.36 (18)	C16—C15—H15A	109.9
C3—N3—H4A^ii^	160.8 (6)	N14—C15—H15B	109.9
C3—N3—H4B^i^	83.4 (5)	C16—C15—H15B	109.9
H4A^ii^—N3—H4B^i^	85.9 (7)	H15A—C15—H15B	108.3
C5—N4—Cu2	109.61 (10)	N17—C16—C15	107.60 (16)
C5—N4—H4A	109.2 (15)	N17—C16—H16A	110.2
Cu2—N4—H4A	109.7 (14)	C15—C16—H16A	110.2
C5—N4—H4B	111.2 (17)	N17—C16—H16B	110.2
Cu2—N4—H4B	112.3 (17)	C15—C16—H16B	110.2
H4A—N4—H4B	105 (2)	H16A—C16—H16B	108.5
N4—C5—C6	108.28 (14)	C18—N17—C16	111.46 (15)
N4—C5—H5A	110.0	C18—N17—Cu2	115.52 (12)
C6—C5—H5A	110.0	C16—N17—Cu2	105.30 (10)
N4—C5—H5B	110.0	C18—N17—H17	111.6 (15)
C6—C5—H5B	110.0	C16—N17—H17	107.8 (15)
H5A—C5—H5B	108.4	Cu2—N17—H17	104.6 (15)
N7—C6—C5	108.34 (14)	N17—C18—H18A	109.5
N7—C6—H6A	110.0	N17—C18—H18B	109.5
C5—C6—H6A	110.0	H18A—C18—H18B	109.5
N7—C6—H6B	110.0	N17—C18—H18C	109.5
C5—C6—H6B	110.0	H18A—C18—H18C	109.5
H6A—C6—H6B	108.4	H18B—C18—H18C	109.5
			
N4—C5—C6—N7	−53.1 (2)	C18—N17—C16—C15	174.49 (16)
N14—C15—C16—N17	−53.0 (2)	C8—N7—Cu2—N4	−148.13 (15)
C8—N7—C6—C5	174.98 (15)	C18—N17—Cu2—N14	−148.74 (14)

Symmetry codes: (i) *x*−1, *y*−1, *z*; (ii) −*x*+1, −*y*+1, −*z*+2.

### (II) [(N-Methylethylenediamine-κ2N,N')copper(II)]-µ2-cyanido-κ2C:N-[bis(cyanido-κC)copper(I)] monohydrate Hydrogen-bond geometry (Å, º)

**Table:**

*D*—H···*A*	*D*—H	H···*A*	*D*···*A*	*D*—H···*A*
O1—H1···N2	0.68 (3)	2.13 (3)	2.810 (3)	171 (3)
N14—H14*A*···O1^iii^	0.83 (2)	2.14 (2)	2.965 (3)	177 (2)
N4—H4*A*···N3^ii^	0.86 (2)	2.26 (2)	3.094 (2)	161.1 (19)
N7—H7···N2^iv^	0.810 (19)	2.460 (19)	3.162 (2)	145.7 (16)
N4—H4*B*···N3^iv^	0.78 (2)	2.53 (2)	3.302 (3)	170 (2)

Symmetry codes: (ii) −*x*+1, −*y*+1, −*z*+2; (iii) −*x*+1, −*y*, −*z*+1; (iv) *x*+1, *y*+1, *z*.
